# Psychometric properties of the DISABKIDS Chronic Generic Module (DCGM-37) when used in children undergoing treatment for cancer

**DOI:** 10.1186/1477-7525-8-109

**Published:** 2010-09-28

**Authors:** Margareta af Sandeberg, Eva M Johansson, Peter Hagell, Lena Wettergren

**Affiliations:** 1 Department of Neurobiology, Care Sciences and Society, Karolinska Institutet, Huddinge, Sweden; 2 Department of Pediatric Oncology, The Karolinska University Hospital, Stockholm, Sweden; 3 Department of Medicine, Karolinska Institutet, and Red Cross College University, Stockholm, Sweden; 4 Department of Health Sciences, Lund University, Sweden

## Abstract

**Background:**

The aim was to evaluate data quality and psychometric properties of an instrument for measurement of health-related quality of life: DISABKIDS Chronic Generic Module (DCGM-37) used in school-aged children with cancer.

**Methods:**

All school-children diagnosed with cancer in Sweden during a two-and-a-half year period were invited to participate in the study. Analysis was performed on combined data from two assessments, two and-a-half and five months after start of cancer treatment (n = 170). The instrument was examined with respect to feasibility, data quality, reliability and construct and criterion-based validity.

**Results:**

Missing items per dimension ranged from 0 to 5.3 percent, with a majority below three percent. Cronbach's alpha values exceeded 0.70 for all dimensions. There was support for the suggested groupings of items into dimensions for all but six of the 36 items of the DCGM-37 included in this study. The instrument discriminated satisfactorily between diagnoses reflecting treatment burden.

**Conclusions:**

The results indicate satisfactory data quality and reliability of the DCGM-37 when used in children undergoing treatment for cancer. Evaluation of construct validity showed generally acceptable results, although not entirely supporting the suggested dimensionality. Continued psychometric evaluation in a larger sample of children during and after treatment for cancer is recommended.

## Background

It is known that treatment for cancer during childhood may cause physical, social and emotional concerns and thus have an impact on health-related quality of life (HRQOL) [1]. Results from studies that have followed HRQOL in children during cancer treatment reveal emotional distress [2,3], diminished physical function and status [2,4] as well as symptoms related to disease and treatment [4,5] during the first year following diagnosis.

There are only a few available valid instruments for assessment of HRQOL in children and adolescents with cancer. One instrument commonly used among these individuals is the Pediatric Quality of Life Inventory (PedsQL) including a generic scale and a disease-specific cancer module [6]. The generic scale was developed to measure HRQOL in healthy populations as well as patient populations in four dimensions. The disease-specific cancer module consists of 27 items encompassing eight dimensions. Psychometric evaluation of the PedsQL generic and cancer module has shown satisfactory results and the instrument is recommended as an outcome measure in research as well as in clinical practice for assessment of HRQOL [6]. Another instrument used among children with cancer is the revised Memorial Symptom Assessment Scale (MSAS) for children aged seven to 12 years [7]. The MSAS is a self-report questionnaire which assesses presence, frequency, severity and associated distress with established cancer-related symptoms. Evaluation of the reliability and validity of the MSAS has shown that children with cancer as young as seven years can report clinically relevant and consistent information about their symptom experience [7]. Follow-up studies after cancer treatment have commonly used generic and domain specific instruments developed for an adult population such as the Short Form Survey (SF-36) [2], the TNO-AZL Children's Quality of Life (TACQOL) [3] and Hospital Anxiety and Depression scale (HADS) [2]. These instruments also provide validated normative data for adolescents and young adults in the general population [8]. Among the instruments mentioned above only PedsQL include items concerning school.

An instrument which includes social issues is the DISABKIDS Chronic Generic Module (DCGM-37) developed in recent years in collaboration with partners from seven European countries [9]. The instrument was developed for assessment of HRQOL in children and adolescents suffering from chronic conditions and addresses aspects that pertain not to specific conditions but to general aspects from the perspectives of children and adolescents [10]. The DISABKIDS consists of four versions: a self-report version for children, a proxy version for parents and a child and proxy version for those younger than eight years (The DISABKIDS - Smileys measure). The long version children self-report DCGM-37 measures HRQOL in six dimensions (Independence, Physical Limitation, Emotion, Social Exclusion, Social Inclusion, and Treatment). Results from pilot testing of the DCGM-37 in children with different chronic conditions have denoted satisfactory internal consistency for all dimensions with Chronbach's alpha coefficient ranging from 0.70 to 0.87 [10,11]. Construct validity evaluated by factor analysis, as well as convergent and discriminant validity have also shown satisfactory results in pilot testing of the instrument [11].

In a recent national study our research group followed HRQOL, school attendance and social interaction with friends in a cohort of Swedish school-children (n = 101) starting treatment for cancer [12]. The DCGM-37 was chosen for assessment of HRQOL as it included relevant items regarding social issues such as school and friends as well as treatment-related issues and was available in the Swedish language [13]. Participants were assessed twice during the first five months of cancer treatment. The results indicated a diminished HRQOL compared to children with chronic conditions over the study period, with girls rating worse HRQOL than boys [12]. Self-reported HRQOL was positively correlated to days of school attendance. Although these results suggest that the DCGM-37 is useful in children undergoing cancer treatment, it has not been tested regarding its psychometric properties and relevance in this target group before. The aim of this study was to evaluate data quality and psychometric properties of the DCGM-37 among school-aged children with cancer.

## Methods

The DCGM-37 was completed at two assessment points approximately two-and-a-half (T1) and five months (T2) after the start of treatment for cancer. These two assessments are part of a larger study following a cohort of school children regarding social life up to six years after diagnosis.

### Sample

All children in Sweden attending compulsory school grades 1-9 (aged seven to 16 years), newly diagnosed with cancer and starting chemotherapy and/or radiation therapy during the period January 2004 to May 2006 were eligible for inclusion in the study [12]. Children who were scheduled to undergo early stem cell transplantation, children with brain tumors exclusively treated with surgery, and children from families that were not able to speak or read Swedish were excluded. One hundred and forty-five children and adolescents were invited to participate in the larger study including three assessments during the first six months of treatment and 101 participated in all the assessments. The second (T1) and third (T2) assessments included the DCGM-37 or the Smiley version. Two participants did, due to organizational reasons not complete the DCGM-37 at T1; eleven participants completed the Smiley version at T1 and ten at T2. Those who completed the DCGM-37 at T1 (n = 83) and T2 (n = 87) were included in this report.

### DCGM-37

The DCGM-37 self-report version for children was used [10]. Those children not considered able to complete the DCGM-37 were approached with the Smiley version developed for those younger than eight years. As the majority of the participating children filled out the long version (DCGM-37), the results from the Smiley version are not included in this report. DCGM-37 consists of six dimensions: Independence (autonomy and living without impairments), Physical Limitation (functional limitations, perceived health), Emotions (emotional worries and concerns), Social Exclusion (stigma, feeling left out), Social Inclusion (acceptance of others, positive relationships) and Treatment (perceived emotional impact of treatment). Each dimension consists of six items and refers to the four previous weeks. All items have five-grade verbal response options ranging from 1 (never) to 5 (very often). Within each scale, item raw scores are summed and transformed into a scale from 0 (worst possible HRQOL) to 100 (best possible HRQOL), following the standard scoring algorithms of the instrument [10]. Missing values were substituted if all but one of the items within a dimension was responded to, meaning, a person-specific mean score was calculated based on the existing answers [11].

### Procedure

The instructions given to the families emphasized the importance of the DCGM-37 being filled out by the child, with support only if required [12]. Children who met the inclusion criteria and their parents were contacted by the consultant nurse in pediatric oncology to receive oral and written information about the study. Informed consent was obtained from those children willing to participate and their parents. The DCGM-37 along with a study-specific questionnaire and a stamped return envelope were given to each hospitalized participant or sent home to participants who were not hospitalized. Ethical approval was obtained from the Regional Ethical Review Board in Stockholm (03-662, 04-208).

To evaluate feasibility seven consultant nurses in pediatric oncology were asked to give their opinions on the items included in the DCGM-37 in a group session. The consultant nurses were chosen because of their expertise in the field, and they represent all pediatric oncology centers in the country.

### Data analyses

All statistical calculations were conducted using SPSS version 16.0 (SPSS Inc, Chicago, IL). The psychometric analyses are based on pooled data from T1 (n = 83) and T2 (n = 87). Pooling of data is recommended when sample sizes are limited in order to increase precision of estimates [14,15].

Feasibility of the DCGM-37 was examined through the nurses' comments of the items as well as by oral and written comments given by the participating children, adolescents, and their parents.

Data quality was evaluated by examination of the amount of missing item responses [14]. Up to 10% missing responses has been suggested as acceptable [16]. The legitimacy of adding up items to generate total dimension scores was tested by examination of item means, standard deviations and corrected item-total correlation within each dimension. Simple summation of item scores into a total score is considered supported when item means and standard deviations within a dimension are similar and corrected item-total correlation coefficients exceed 0.3 [14]. Furthermore, items within each dimension should represent the same latent variable. This is considered supported if corrected item-total correlations are ≥0.40 [14].

The distribution of dimension scores was examined by calculations of means, standard deviations, and floor and ceiling effects. Cronbach's alpha values were calculated to estimate the internal consistency reliability of the DCGM-37. Floor and ceiling effects should be less than 15% [17] and alpha values ≥0.70 are considered acceptable whereas ≥0.80 are preferred [18].

Multi-trait-scaling analyses with correction for overlap was performed to examine the internal construct validity of the DCGM-37. This is supported when an item's corrected item-total correlation is ≥0.40 with the dimension it is hypothesised to belong to, while correlating weaker with all other dimensions [19,20]. The proportion (%) of items within each dimension that met these criteria was examined and referred to as the scaling success rate.

To examine whether the six dimensions appear to measure different aspects of HRQOL the correlation coefficients between dimensions were compared with each dimension's internal consistency (Cronbach's alpha). If the alpha value of a dimension is higher than the dimension's correlation to the other dimensions, it indicates that dimension scores represent different aspects of HRQOL [14].

To evaluate criterion-based validity the instrument's capacity to discriminate between patients differing in symptom burden, data from children diagnosed with acute lymphoblastic leukemia (ALL) were compared to those with sarcoma. In the pooled DCGM-37 data of 170 children, 50 were undergoing treatment for ALL and 32 for sarcoma. Children with ALL exclusively receive chemotherapy and are often described by physicians and nursing staff as a group with fewer side effects from treatment compared to other diagnoses. Children with sarcomas receive combination therapy including surgery, chemotherapy and radiation therapy and are often described as a group with more side effects and complications than other diagnoses [21]. In line with this our previous report showed that children with osteosarcoma were more likely to be absent from school than those with other diagnoses [12]. Independent t-tests were calculated to investigate potential differences in mean values for the DCGM-37 dimensions by diagnosis (ALL vs. sarcoma) and age groups (7-12 years vs. 13-16 years). Effect sizes (ES) were calculated to highlight the clinical importance of potential mean value differences. According to Cohen [22], ES = 0.20-0.50 indicates a small difference, ES = 0.51-0.80 indicates a medium difference and ES > 0.80 indicates a large difference. P-values ≤ 0.05 were considered statistically significant.

## Results

Socio-demographic and clinical characteristics of the sample are presented in table 1. Due to a negative reaction from parents, one of the items in the DCGM-37 (Item 17: "Do you have fears about the future because of your condition?") was excluded early in the study and treated as a missing value for all participants. After the exclusion of this item the majority of the participants filled out the instrument without reporting any problems. Some parents described that, prior to filling in the questionnaire; they were worried that the items would upset their children and make them doubtful about the future. However, after completing the questionnaire together, some parents expressed appreciation over the way the instrument opened up for deeper conversation with their offspring. Evaluation by seven consultant nurses in pediatric oncology, suggested that all but one item was feasible. Item 30 ('Do your friends enjoy being with you?') was questioned if appropriate in the Swedish culture as it may not be fully accepted to describe oneself in a very positive manner.

**Table 1:** Socio-demographic characteristics of participating children

Participants	T1	T2	Pooled data
Total number, n	83	87	170
			
Sex, n (%)			
Boys	47 (57)	50 (57)	97 (57)
Girls	36 (43)	37 (43)	73 (43)
			
Age at diagnosis, median (range)	12 (7-16)	12 (7-16)	12 (7-16)
			
Age groups, n (%)			
7-12 years	43 (52)	44 (51)	87 (51)
13-16 years	40 (48)	43 (49)	83 (49)
			
School grade at diagnosis, median (range)	6 (1-9)	6 (1-9)	6 (1-9)
			
Siblings living at home, n (%)	73 (88)	77 (88)	150 (88)
			
Diagnoses, n (%)			
Acute lymphoblastic leukaemia	24 (29)	26 (30)	50 (29)
Acute myeloid leukaemia	4 (5)	5 (6)	9 (5)
CNS^a ^tumours	13 (16)	13 (15)	26 (15)
Non - Hodgkin's lymphoma	11 (13)	10 (12)	21 (12)
Hodgkin's Lymphoma	8 (10)	9 (10)	17 (10)
Neuroblastoma	0	1 (1)	1 (1)
Sarcoma	16 (19)	16 (18)	32 (19)
Rhabdomyosarcoma	4 (5)	4 (5)	8 (5)
Other^b^	3 (3)	3 (3)	6 (4)

^a ^Central nervous system

^b ^Germ cells tumor, Soft tissue sarcoma (nerve), Sertoli leydig cell tumor, Synovial sarcoma, Teratoma and a mixed tumor

The percentage of missing items by dimension was below six percent (range 0-5.3), the largest number of missing items being found in the dimension Social Exclusion (Table 2). Reasons for not responding to an item were seldom reported. The reason for not answering items regarding school was occasionally explained by the statement, "I have not been to school". Items not answered in the Treatment dimension were in some cases explained by the statement "I am not taking any medication".

**Table 2:** Descriptive and psychometric statistics for the DCGM-37 in Swedish children on cancer treatment, pooled data, n = 170

Dimensions	n	Mean (SD)	Missing items, range (%)	Ranges of item mean (SD)	Floor/Ceiling effect (%)	Reliability (α)	Item-to-own dimension correlation (range)	Item-to-other dimension correlation (range)	**Scaling success (%) **^**a**^
Independence	170	60.4 (19.5)	0-4 (0)	3.03-3.89 (0,96-1,18)	0.6/0	0.81	0.43-0.68	0.15-0.60	93
Physical Limitation	169	53.1 (19.6)	0-2 (0.6)	3.25-3.94 (1,02-1,34)	0/0	0.76	0.32-0.66	0.19-0.58	87
Emotion	165	58.5 (19.9)	3-5 (2.9)	2.88-3.53 (0,97-1,22)	0/1.2	0.84	0.54-0.70	0.25-0.65	100
Social exclusion	161	68.5 (17.7)	1-13 (5.3)	3.31-4.51 (0,68-1,22)	0/2.9	0.76	0.35-0.63	0.09-0.60	90
Social inclusion	168	61.9 (17.3)	0-6 (1.2)	3.02-4.35 (0,83-1,16)	0/0.6	0.71	0.28-0.66	-0.01-0.65	73
Treatment	164	64.0 (25.8)	4-8 (3.5)	3,35-4.02 (1,14-1,60)	2.4/10.0	0.87	0.54-0.77	0.14-0.47	100

^a ^Number of item-to-other dimension correlations that are stronger than the corrected item-total correlation within a dimension/Total number of discriminant validity tests (i.e., number of items × number of dimensions minus 1), expressed as a percentage.

Item means and standard deviations within the respective dimensions were roughly equivalent (Table 2). All but one corrected item-total correlation exceeded 0.30. The corrected item-total for item 31 in the dimension Social Inclusion was 0.28, and in all but six instances (items 10, 11, 22, 26, 30, 31) the item-total correlation was ≥0.40 (Table 2, Table 3).

**Table 3:** Multitrait-scaling analysis of the DCGM-37, pooled data (n = 170)

	Item	(1)	(2)	(3)	(4)	(5)	(6)
(1) Independence	1	**0.43 **^a^	0.29	0.39	0.35	0.34	0.23

	2	**0.56**	0.36	0.48	0.38	0.48	0.38

	3	**0.66**	0.59	0.48	0.41	0.56	0.17

	4	**0.63**	0.49	0.51	0.52	0.46	0.23

	5	**0.68**	0.60	0.53	0.46	0.55	0.20

	6	**0.50**	0.55 ^d^	0.37	0.31	0.58 ^d^	0.15

(2) Physical Limitation	7	0.57	**0.50**	0.36	0.36	0.58 ^d^	0.20

	8	0.49	**0.66**	0.57	0.51	0.49	0.22

	9	0.55	**0.58**	0.53	0.47	0.54	0.25

	10	0.29	**0.32 **^c^	0.31	0.34 ^d^	0.36 ^d^	0.31

	11	0.26	**0.37 **^c^	0.33	0.40 ^d^	0.28	0.19

	12	0.49	**0.59**	0.48	0.54	0.51	0.28

(3) Emotion	13	0.43	0.39	**0.63**	0.57	0.25	0.41

	14	0.52	0.51	**0.70**	0.59	0.41	0.42

	15	0.41	0.47	**0.60**	0.51	0.43	0.25

	16	0.37	0.38	**0.56**	0.44	0.33	0.34

	18	0.62	0.62	**0.67**	0.65	0.51	0.39

	19	0.40	0.48	**0.54**	0.42	0.46	0.40

(4) Social Exclusion	20	0.55	0.55	0.60	**0.63**	0.53	0.32

	21	0.27	0.35	0.35	**0.45**	0.23	0.34

	22	0.30	0.43 ^d^	0.42 ^d^	**0.34 **^c^	0.30	0.09

	23	0.29	0.31	0.37	**0.42**	0.32	0.29

	24	0.33	0.43	0.48	**0.53**	0.34	0.25

	25	0.50	0.46	0.60 ^d^	**0.51**	0.40	0.31

(5) Social Inclusion	26	0.34 ^d^	0.30	0.39 ^d^	0.42 ^d^	**0.31 **^c^	0.17

	27	0.52	0.56	0.36	0.35	**0.66**	0.21

	28	0.62 ^d^	0.61 ^d^	0.44	0.39	**0.60**	0.25

	29	0.56 ^d^	0.65 ^d^	0.43	0.38	**0.52**	0.31

	30	0.26	0.21	0.21	0.31 ^d^	**0.31 **^c^	-0.01

	31	0.29 ^d^	0.26	0.24	0.16	**0.28 **^b, c^	0.26

(6) Treatment	32	0.21	0.27	0.31	0.25	0.26	**0.49**

	33	0.16	0.29	0.40	0.37	0.22	**0.69**

	34	0.36	0.37	0.41	0.42	0.35	**0.54**

	35	0.14	0.21	0.39	0.29	0.16	**0.77**

	36	0.25	0.22	0.35	0.24	0.22	**0.77**

	37	0.34	0.37	0.47	0.39	0.35	**0.71**

^a^Corrected item-total (item-to-item-within-own-dimension) correlations are in bold.

^b^Corrected item-total correlations failing to meet the ≥0.30 criterion

^c^Corrected item-total correlations failing to meet the ≥0.40 criterion

^d^Item-to-other-dimension correlation exceeding the corrected item-total correlation (scaling failure)

Floor effects ranged between 0-2.4% (Table 2). Similarly, ceiling effects were between 0-2.9% with exception for the Treatment dimension, which had a larger, still acceptable effect of 10% (Table 2). Reliability for all dimension scores exceeded the recommended criteria minimum of 0.70 and three exceeded the preferred value of 0.80 (Table 2).

Multi-trait scaling analyses supported the grouping of items into dimensions for 26 of the 36 items as their corrected item-total correlations exceeded the correlations with other dimension scores; scaling success rates ranging between 73-100% (Table 2, Table 3).

Internal consistencies for all dimensions were larger than their correlation coefficients to the other dimensions (Table 4). This suggests that the six dimension scores represent distinct constructs. However, the alpha values for the Social Inclusion dimension (0.71) was only marginally above this dimension's correlation with the Independence (0.68) and Physical Limitation dimensions (0.69), suggesting some overlap. Similarly, the alpha value for the Social Exclusion dimension (0.76) was relatively close to its correlation with the Emotion dimension (0.71).

**Table 4:** Inter-dimension correlations for DCGM-37, pooled data, n = 170

	Independence	Physical Limitation	Emotion	Social Exclusion	Social Inclusion	Treatment
Independence	(0.81) ^a^					
Physical Limitation	0.67	(0.76)				
Emotion	0.64	0.63	(0.84)			
Social Exclusion	0.58	0.65	0.71	(0.76)		
Social Inclusion	0.68	0.69	0.55	0.52	(0.71)	
Treatment	0.31	0.36	0.50	0.41	0.33	(0.87)

^a ^Internal consistency reliability (Cronbach's alpha) is presented in parenthesis in the diagonal

Mean values by age groups (7-12 years vs. 13-16 years) are presented in Table 5. HRQOL did not differ between the two age groups with one exception, Physical Limitation was rated significantly higher among the 13-16-years-olds compared to the younger group. Effect sizes were all low or medium (Table 5).

**Table 5:** Differences in self-reported HRQOL between age groups: 7-12 years (n = 87) and 13-16 years (n = 83), pooled data

	7-12 years	13-16 years		
DCGM-37 dimensions	**Mean**^**a **^**(SD)**	**Mean**^**a **^**(SD)**	P	**ES **^**b**^
Independence	57.8 (19.5)	63.2 (19.2)	ns	0.28
Physical Limitation	50.0 (18.7)	56.3 (20.0)	< 0.05	0.33
Emotion	57.8 (19.9)	59.3 (20.0)	ns	0.08
Social Exclusion	68.9 (17.7)	68.1 (17.8)	ns	0.05
Social Inclusion	59.8 (16.7)	64.2 (17.8)	ns	0.25
Treatment	60.3 (26.0)	67.9 (25.1)	ns	0.30

^a ^Scores range from 0 to 100, higher scores represent a better HRQOL Differences tested by student's unpaired *t*-test.

^b^Effect size

ns, non-significant

Mean value differences and effect sizes between ALL and sarcoma are presented in Table 6. Patients with sarcoma scored significantly lower than those with ALL in all dimensions. Effect sizes were all large (Table 6).

**Table 6:** Significant differences in self-reported HRQOL between children on cancer treatment for Acute lymphoblastic leukaemia (ALL) (n = 50) and Sarcoma (n = 32), pooled data

	ALL	Sarcoma		
DCGM-37 dimensions	**Mean**^**a **^**(SD)**	**Mean**^**a **^**(SD)**	P	**ES **^**b**^
Independence	66.3 (14.9)	44.0 (20.7)	< 0.001	1.25
Physical Limitation	56.9 (18.1)	37.4 (16.1)	< 0.001	1.14
Emotion	62.6 (18.5)	43.3 (14.2)	< 0.001	1.18
Social Exclusion	74.6 (16.7)	52.8 (17.7)	< 0.001	1.27
Social Inclusion	62.4 (15.2)	48.3 (14.8)	< 0.001	1.22
Treatment	64.2 (23.8)	44.1 (22.3)	< 0.001	0.87

^a ^Scores range from 0 to 100, higher scores represent a better HRQOL

Differences tested by student's unpaired *t*-test

^b^Effect size

## Discussion

This study assessed the data quality and psychometric properties of the DCGM-37 in a sample of Swedish school-aged children receiving treatment for cancer. The instrument appears to be a feasible instrument with satisfactory data quality and generally acceptable psychometric properties in children undergoing treatment for cancer.

Data quality was satisfying with overall acceptable amount of missing values. Missing responses were primarily associated with the two school-related items, mostly due to school absence. Missing data in the Treatment dimension was sometimes due to the respondent not taking any medication at the time of completing the questionnaire.

Summation of item scores into dimension scores without standardization or weighting was supported by similarity of means and standard deviations within dimensions and item-total correlations exceeding 0.3 [14]. Similarly, reliability was acceptable or satisfying and there were no abundant ceiling or floor effects. This provides indirect support for the instrument's sensitivity and responsiveness [17]. Accordingly, all dimension scores were able to discriminate between children with ALL and sarcoma. We also scrutinized our results to corresponding results collected from field studies of the instrument to be able to make comparisons to other diagnoses. Children undergoing treatment for cancer rated their HRQOL as poorer in all dimensions compared to children with other chronic conditions [11]. It is promising that still, the DCGM-37 was able to discriminate patients with regard to treatment burden.

However, data also challenged the interpretability of some dimension scores as item-total correlations in three dimensions (Physical Limitation, Social Exclusion, Social Inclusion), were below 0.4, which is considered to suggest that an item may not represent the same construct as the dimension [14,16]. Furthermore, scaling success rates for all dimensions but Emotion and Treatment indicated that the grouping of items into dimensions may not be optimal. With one exception (item 6) scaling failure involved the Social Exclusion and Social Inclusion dimensions.

Considering the six problematic items from the multi-trait-scaling analyses, one may speculate on possible explanations for these findings in relation to the population of children undergoing cancer treatment. It appears that the dimensionality regarding Physical Limitation and the social dimensions (Social Inclusion, Social Exclusion) are difficult to separate from one another. It is reasonable that physical health and social life are related to one another in persons undergoing heavy treatment. Three of these items (item 10, 26, 31) concern the child's perception of explaining the disease to others. The result may represent a mixture of effects of cancer treatment on all dimensions. Reasons for not communicating with peers may be a result of physical weakness giving social restrictions and be related to both physical and social aspects. The results regarding sleep difficulty (item 11) and problems concentrating (item 22) may reveal the same physical/social interaction. Item 30 ('Do your friends enjoy being with you?') in the dimension Social Inclusion, showed a moderate correlation coefficient to Independence as well as to Emotion and Social Exclusion. It is conceivable that this item implies emotional worries and concerns as well as the feeling of being left out, which may explain the observed dimensional ambiguity related to this item. Despite indications of scaling failure due to the items discussed above, we consider them to be of great interest in a population of children and adolescents with cancer [5]. The question that our observations raise, however, is whether they are suitable representations of the dimensions that they are suggested to belong to.

There are some limitations in the present study. The sample size is considered small and includes heterogeneous cancer diagnoses. This is difficult to rectify as the Swedish pediatric population diagnosed with cancer is, for natural reasons, small. The authors of this paper suggests continued psychometric evaluation of DCGM-37 in a larger sample of children with cancer, preferably in a Nordic multicenter study, including more conclusive analyses such as Rasch, item-response theory or confirmatory factor analyses. A strength of the present study is, however, that it is a nationwide study including all children in Sweden diagnosed with cancer during a two-and-a-half year period and that time for assessments in relation to start of treatment is about the same for all participants.

## Conclusions

The results provided in this paper support feasibility, and data quality as well as general support for the psychometric properties of the DCGM-37 when used in children undergoing treatment for cancer. However the dimensionality of the instrument is uncertain which may impact score interpretability. Further psychometric evaluation in a large sample of children with cancer as well as after pediatric cancer treatment is recommended to better understand these aspects and provide firmer conclusions.

## Competing interests

The authors declare that they have no competing interests.

## Authors' contributions

Conception and design: MafS, LW and PH. Collection and assembly of data: MafS. Data analysis and interpretation: MafS, LW and PH. First draft of the manuscript: MafS. Critical revision: MafS, LW, PH and EJ. All authors read and approved the final manuscript.
